# Nutritional Status, Body Composition, and Inflammation Profile in Older Patients with Advanced Chronic Kidney Disease Stage 4–5: A Case-Control Study

**DOI:** 10.3390/nu14173650

**Published:** 2022-09-03

**Authors:** Mar Ruperto, Guillermina Barril

**Affiliations:** 1 Department of Pharmaceutical & Health Sciences, School of Pharmacy, Universidad San Pablo-CEU, CEU Universities, Urbanización Monteprincipe, 28660 Madrid, Spain; 2 Nephrology Department, Hospital Universitario La Princesa, C/Diego de León 62, 28006 Madrid, Spain

**Keywords:** advanced chronic kidney disease, body composition, inflammation, Mediterranean cohort, nutritional status, older adults, overweight, obesity, predialyisis care, prognostic nutritional index, s-albumin

## Abstract

Nutritional status is a predictor of adverse outcomes and mortality in patients with advanced chronic kidney disease (ACKD). This study aimed to explore and evaluate risk factors related to nutritional status, body composition, and inflammatory profile in patients with ACKD compared with age- and sex-matched controls in a Mediterranean cohort of the Spanish population. Out of 200 volunteers recruited, 150 participants (64%) were included, and a case-control study was conducted on 75 ACKD patients (stages 4–5), matched individually with controls at a ratio of 1:1 for both age and sex. At enrolment, demographic, clinical, anthropometric, and laboratory parameters were measured. Bioimpedance analysis (BIA) was used to assess both body composition and hydration status. ACKD patients had lower body cell mass (BCM%), muscle mass (MM%) phase angle (PA), s-albumin, and higher C-reactive protein (s-CRP) than controls (at least, *p* < 0.05). PA correlated positively with BCM% (cases: *r* = 0.84; controls: *r* = 0.53, *p* < 0.001), MM% (cases: *r* = 0.65; controls: *r* = 0.31, *p* < 0.001), and inversely with s-CRP (cases: *r* = −0.30, *p* < 0.001; controls: *r* = −0.31, *p* = 0.40). By univariate and multivariate conditional regression analysis, total body water (OR: 1.186), extracellular mass (OR: 1.346), s-CRP (OR: 2.050), MM% (OR: 0.847), PA (OR: 0.058), and s-albumin (OR: 0.475) were significantly associated among cases to controls. Nutritional parameters and BIA-derived measures appear as prognostic entities in patients with stage 4–5 ACKD compared to matched controls in this Mediterranean cohort.

## 1. Introduction

The world’s population is aging, with those over 65 years of age being the largest and fastest-growing age group. One out of four people could be over the age of 65 in the forthcoming decades, and those over 80 years of age are also expected to triple, from 143 million in 2019 to 426 million in 2050 [1].

Chronic kidney disease (CKD) is a common and widespread condition affecting more than 10% of the world’s population and is projected to become the fifth leading cause of death by 2040 [2]. Contributing risk factors, such as advanced age, diabetes mellitus (DM), and cardiovascular disease (CVD), have been identified as the most important causes leading to CKD, with an estimated prevalence of 27.9% to 44% in aged 70 years or over [2].

Older adults are particularly vulnerable to nutritional and dietary disorders. Some of the attributable aging factors, such as functional status, reduced sensory impairment and appetite, inadequate food intake, polypharmacy, and comorbid conditions, are either individual or jointly associated with worse nutritional status among older adults [3]. Malnutrition is fairly low at about 3.30% in elderly Spaniards without CKD living in the community [4], whilst in older CKD patients, the so-called protein-energy wasting (PEW) syndrome has been reported in 11–54% of clinical studies [5,6,7]. Several significant factors affect nutritional and metabolic status in CKD, such as advanced age and uremia-induced disturbances, including anorexia, metabolic acidosis, systemic inflammation, and comorbidities (CVD, DM) leading to PEW syndrome and adverse outcomes. In a previous study of older adults with stages 4–5 CKD, nutritional disorders were found in more than one-third of the patients in the first year of follow-up [7]. The predialysis patient’s records (PREPARE-2) study [6], conducted on 376 ACKD patients reported that age 75 years or over, female, and having BMI < 25 kg/m^2^ were significant independent risk factors for PEW. Thus, a prospective multicenter European study in non-CKD older adults and stage 3b-4 CKD patients aged 75 years and over [8] showed that median BMI was significantly associated with overweight and higher central abdominal fat in both men and women. Among the commonly used anthropometric measures, BMI is the most widely used indicator of body fatness. Higher BMI (≥25 kg/m^2^), due to a phenomenon known as the “obesity paradox”, was considered a survival factor in stage 3–5 CKD in some studies [9,10]. Nevertheless, the protective effects of overweight or obesity in CKD are dependent both on body composition (ratio of fat mass to muscle mass) and abdominal adiposity as classical risk factors.

Abnormal fat depots measured by anthropomorphic measures such as waist circumference (WC) and conicity index have been shown to be independent predictors of increased CVD risk and all-cause mortality [11,12,13,14]. Studies suggested that abdominal fat could be predicted risk more accurately when a combination of BMI and WC measurement was used [11,15].

Bioelectrical impedance analysis (BIA) is a non-invasive and easy-to-perform method that more objectively assesses both body composition such as muscle, fat, and fluid distribution between compartments. Indeed, BIA can be used as a guide to monitor changes in body composition and between body fluids [16,17]. In the ACKD state, the inability to concentrate or dilute urine and the need to eliminate solute load and free water are primarily responsible for water balance disorders or overhydration.

S-albumin, an acute-negative phase reactant is one of the stronger predictors of adverse outcomes and mortality in CKD and dialysis patients [18,19]. Hypoalbuminaemia is associated with superimposed conditions such as inflammation and overhydration, leading to an increased risk of morbidity and mortality in CKD patients [18]. In addition, chronic low-grade inflammation as measured by inflammatory markers such as serum C-reactive protein (s-CRP) or interleukin-6, may stimulate changes in s-albumin, body composition, and increased muscle wasting in older ACKD patients [20].

Nutritional assessment and monitoring, along with nutritional and inflammatory markers, are particularly important in the preventive and therapeutic approach to help slow down CKD progression and ameliorate or reverse nutritional disturbances. Although several methods are available for early detection and nutritional assessment, including validated nutritional screening tools (malnutrition-inflammation score, subjective global assessment, mini-nutritional assessment), some biochemical parameters (s-albumin, s-CRP), as well as BIA-derived measurements (phase angle, body cell mass, extracellular body water, total body water, etc.) [21], in practice, their use and interpretation in isolation, do not provide a complete nutritional diagnosis in CKD patients in clinical settings.

The prognosis nutritional index (PNI) is a simple novel indicator of the nutritional and immune status which consists of s-albumin and total lymphocyte count [22]. The PNI reflects the balance between the inflammatory status and impaired protein synthesis secondary to CKD derangements. Several previous studies [23,24,25,26,27] have shown that the significance of PNI is associated with poor clinical outcomes and predicts survival in a variety of solid tumors and some other disease conditions. A recent study in patients with stage 3–4 CKD in adults over 80 years [28] concluded that PNI was a significant predictor of mortality as well as a useful prognostic indicator of nutritional disturbances.

Despite the well-documented clinical importance of nutritional status in the management of elders with CKD, refs. [6,7,8,28] few studies are available in patients with stages 4–5 CKD in older adults prior to the initiation of dialysis. Based on available studies, the burden of CKD and nutritional risk factors remain inconclusive regarding the non-CKD elderly population. Therefore, this case-control study aimed to explore and evaluate risk factors related to nutritional status, body composition, and inflammatory profile in patients with ACKD compared with age- and sex-matched controls in a Mediterranean cohort of the Spanish population.

## 2. Materials and Methods

### 2.1. Patient Population

A sample of two hundred volunteers recruited from the advanced chronic kidney disease unit (ACKD unit) of the Hospital Universitario de La Princesa, (Madrid, Spain) for the period September 2008 to December 2018 (cases and controls) was invited to participate. Cases were collected from the ACKD unit while controls were enrolled from the community. Inclusion criteria for case selection were age equal or over 65 years; stages 4–5 CKD (estimated-glomerular filtration rate (e-GFR): ≤30 mL/min/1.73 m^2^) and follow-up in ACKD unit higher than 3 months.

Controls were selected for the study if they showed agreement to participate, were aged over 65 years, and absence of CKD or any other pathology influencing nutritional and inflammatory status. Cases and controls were excluded if they had advanced heart failure, chronic lung disease, cirrhosis, malignancies, or bacterial infections; amputation of a limb or pacemaker users; oral nutritional supplements (ONS) or any additional nutritional support (enteral or parenteral nutrition); corticosteroid therapy; hospital admission or surgery of any cause with an impact on nutritional status, inflammation, and body composition in the last 3 months; any pathology with life expectancy < 3 months.

**Figure 1:**
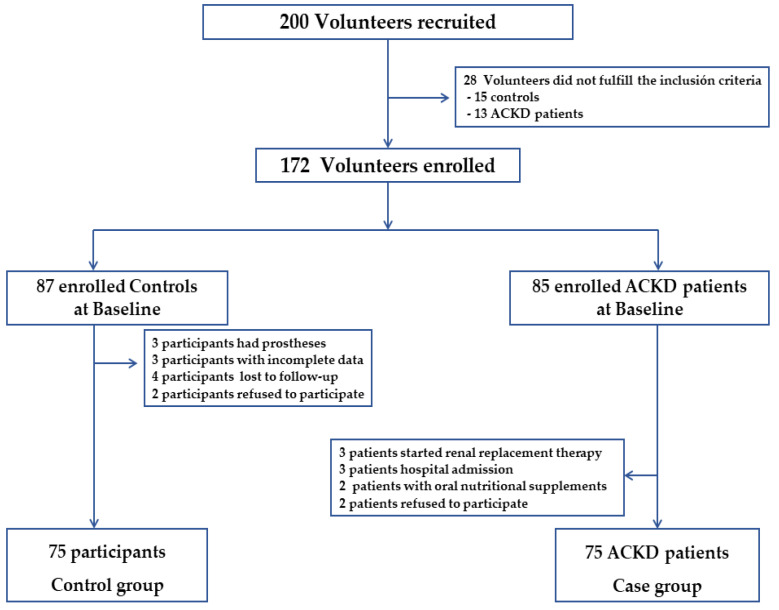
Flowchart of participants who met inclusion and exclusion criteria for the study population. ACKD, advanced chronic kidney disease.

A total of 172 volunteers were initially enrolled to participate in the study. The previous sample elected in the study was 87 controls and 85 patients with ACKD (cases) (Figure 1). Twelve volunteers from the control group and 10 ACKD patients were excluded. Out of 200 volunteers recruited, 150 participants (64%) were included, and a case-control study was conducted on 75 ACKD patients (stages 4–5), matched individually with controls at a ratio of 1:1 for both age and sex. This study was carried out by the Helsinki Declaration and Good Clinical Practice Guidelines. Ethical approval and permission were obtained from the local Ethics Committee and written informed consent was signed by all participants before starting the study (code no. 2649).

### 2.2. Data Collection

Sociodemographic and clinical data were examined from the medical records of all participants. The etiology of CKD, and nutritional and laboratory parameters of each participant were collected. The estimated glomerular filtration rate (eGFR) was measured by the Chronic Kidney Disease Epidemiology Collaboration (CKD-EPI) using 2021-CKD-EPI Creatinine equation [29] as follows: eGFR_cr_ = 142 × min(S_cr_/κ, 1)^α^ × max(S_cr_/κ, 1)^−1.200^ × 0.9938^Age^ × 1.012 [if female], where S_cr_ is standardized serum creatinine expressed in mg/dL; κ is a constant 0.7 (females) or 0.9 (males); α = −0.241 (female) or −0.302 (male) and min-max (S_cr_/κ, 1) is indicating the minimum or maximum of S_cr_/κ or 1.0 and, age is expressed in years.

The eGFR was classified according to the Kidney Disease Improving Global Outcomes (KDIGO) guidelines [30] in ACKD patients (cases).

### 2.3. Anthropometric Measurements

Anthropometric parameters such as body weight (BW), standard body weight (SBW), height, body mass index (BMI), and triceps skinfold thickness (TST) were performed with standardized methods. The percentage of SBW was calculated as follows: SBW (%) = (BW/IBW) × 100, where the BW was the patient’s body weight and the IBW was the ideal body weight of Spanish people of the same, sex, height, and age range [31]. BMI was calculated as body weight (kg) divided by squared height (m^2^). A BMI of 25–29.9 kg/m^2^ was set for overweight and BMI ≥ 30 kg/m^2^ for obesity [32]. Triceps skinfold thickness (TST) was performed by Lange Skin Calipers (Cambridge Instruments, Cambridge, MD, USA) using standard techniques. Mid-arm circumference (MAC) was measured at the midpoint of the non-dominant or arteriovenous fistula-free arm with an inextensible tape measure. Mid-arm muscle circumference (MAMC) was calculated as follows: MAMC (cm) = MAC (cm) − 0.314 × TSF (mm). The percentage of MAMC (%) and TST (%) was compared with anthropometric reference values at the 50th percentile for sex, age, and height in the older Spanish population [31].

Waist circumference (WC) was measured according to WHO guidelines at the mid-point between the lower border of the rib cage and the iliac crest using a rubber measuring tape by validated methods [33]. The cut-off point of WC ≥ 102 cm for males and WC ≥ 88 cm for females were set on a CV risk factor.

The conicity index was calculated using the equation proposed by Valdez [34] which includes the measures of WC, weight, and height: WC/[0.109 × square root of (weight/height)]. The conicity index is based on estimating the volume of the human body constructed to oscillate between the shapes of a cylinder and a double cone, assuming a constant body density [34,35]. A conicity index value of 1.50 means that the individual’s WC is 1.5 times larger than the circumference of a hypothetical cylinder, and the expected theoretical range is 1 to 1.73.

### 2.4. Analysis of Body Composition

Body composition analysis was performed using a single-frequency bioelectrical impedance (BIA) device (BIA-101^®^. Akern-RJL Systems, Florence, Italy) that employs a constant electrical flow at 800 mA through the human body in decubitus supine. One pair of disposable electrodes (BiatrodesTM 1000S. Akern. Florence, Italy) was placed on the dorsum of the right hand over the third metacarpophalangeal joint and wrist, and the second pair was placed over the ipsilateral joints of the third metacarpophalangeal joint and right ankle.

Hydration status was measured by using certain parameters such as total body water (TBW), extracellular water (ECW), intracellular water (ICW), and exchangeable Na/K. Body cell mass (BCM), extracellular mass (ECM), fat-free mass (FFM), muscle mass (MM), and fat mass (FM) were used as body composition measurements (all expressed in both, kg or %). Phase Angle (PA) was used as an indicator of cellular health and integrity. All bioimpedance-derived measurements were calculated using the Bodygram Pro v.3.0 software^®^. (BIA-101®.Akern-RJL Systems, Florence, Italy) BIA has previously been used as a usual technique for CKD patients [36], and controls [37].

### 2.5. Laboratory Parameters

Blood samples were obtained from all participants and frozen at −80 °C for subsequent automated analysis of all samples. Laboratory data included total cholesterol, serum creatinine (s-creatinine), total proteins, s-calcium, s-phosphorous, s-potassium as well as hemoglobin and total lymphocytes count by using standard automated analyzers (Abbot, Aeroset^®^, Diamond Diagnosis, Holliston, MA, USA). Serum albumin (s-Albumin) was analyzed by using the bromocresol green method and a non-high sensitivity serum C-reactive protein (s-CRP) was measured by immunoturbidimetry assay (Roche/Hitachi 904^®^/Model P: ACN 218, Roche Diagnostics, Basel, Switzerland).

### 2.6. Prognosis Nutritional Index

The prognostic nutritional index (PNI) is an easy-to-use score that has previously been used in the elderly with CKD [28,38] and dialysis patients [39,40,41,42]. The Onodera’s PNI [22] was calculated as follows: (10 × serum albumin (g/dL)) + (0.005 × total lymphocyte count). In agreement with a previous study in elderly CKD patients [28], the cut off-point of PNI was set at 39 points, where a high PNI was ≥39 points (no nutritional risk) and a low PNI was <39 points (nutritional risk). This immune-nutritional score was used to assess nutritional risk within the case and control groups.

### 2.7. Statistical Analysis

Inclusion and cleaning of the data in the database, processing, and statistical analysis were carried out in the last quarter of 2020 and during 2021. Data were analyzed using descriptive statistics, such as median, mean and standard deviation for continuous variables and frequency for categorical variables. The normality of the variables was checked in the preliminary analysis of the data and the central limit theorem was applied, assuming a sample size >30 subjects and by the homogeneity of variances. To compare differences between cases and controls, *p*-values were calculated using Chi-square and Fisher’s exact test for categorical variables and Student’s *t*-test for continuous variables. Pearson’s Chi-square parametric correlations were examined to assess the strength of the association between the variables. Univariate and multivariate conditional logistic regression analyses were used, and the corresponding odds ratio (OR) and 95% confidence interval (95%CI) were calculated. Only data from the univariate analysis that had convergence between cases and controls and a *p*-value < 0.20 were shown. For the conditional multivariate regression analysis using cases and controls as the dependent variable, only those variables with a *p*-value of 0.10 or less were included in the regression model. To test for potential confounders in the multivariate conditional regression model, the direction of association was explored using Pearson’s Chi-square parametric correlations together with collinearity and the change of more than 10% in the coefficient (the OR) of the variable(s) included in the model as methods to control for confounders. Statistical analyses were conducted on both SPSS v. 27.0 (BIA-101^®^.Akern-RJL Systems, Florence, Italy) and Stata 14.1 statistical software (StataCorp LLC, CA, USA).

## 3. Results

### 3.1. Global Data and Comparison between Cases and Controls

This case-control study included 150 older adults (75 ACKD patients and 75 controls) matched by age and sex. The main etiology leading to CKD among cases [*n* = 15 (20.00%)] and controls [*n* = 7 (9.33%); *p* > 0.001] was DM. Table 1 summarizes the demographic and clinical characteristics of the study. No mean differences were observed for BW and SBW, whilst a significant difference was found for BMI (cases: 26.58 ± 4.64 vs. controls: 28.73 ± 6.28; *p* < 0.001).

Controls had globally more overweight and obesity (*n* = 57; 76%) that ACKD patients (*n* = 45; 60%) (*p* = 0.005). Analyzing WC by sex as a CV risk factor, 61 females presented a high frequency of WC ≥ 88 cm (ACKD: 43.24%; controls: 39.10%; *p* = 0.46) while high frequencies were also found with the cut-off point of WC ≥ 102 cm proposed in 31 males (ACKD: 17.10%; controls: 23.70%; *p* = 0.75) (data not showed). High mean conicity index values were obtained with WC ≥ 88 cm in either control females (1.41 ± 0.12) or females with ACKD (1.38 ± 0.05) (*p* = 0.146). Relatedly, in 31 men with WC ≥ 102 cm, mean conicity index values were found high in both controls (1.44 ± 0.10) and patients with ACKD (1.46 ± 0.10) (*p* = 0.673). Similarly, elevated mean values of TST% and MAMC% were found, although the results remained non-significant when the case-control comparison was made (Table 1).

**Figure 2:**
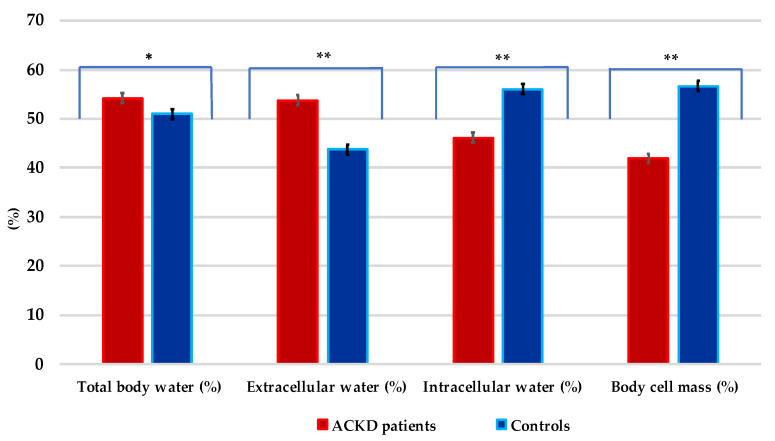
Distribution of the hydration status in patients with advanced chronic kidney disease and controls. * *p* = 0.006; ** *p* < 0.001. Values are expressed in percentages (%).

As shown in Figure 2, TBW%, ECW%, ICW%, and BCM% differed significantly between cases and controls. Higher mean values of ECW, ECM, and exchangeable Na/K were found, while ICW% and BCM% showed to be lower in patients with ACKD than in controls (all, *p* < 0.001).

**Figure 3:**
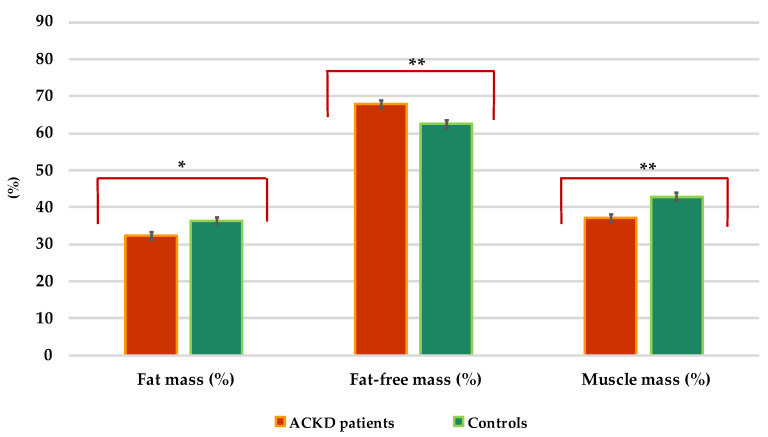
Distribution of the body composition parameters in patients with advanced chronic kidney disease and controls. * *p* = 0.006, ** *p* < 0.001. Values are expressed in percentages (%).

Cases had significantly lower mean values of BCM, FM, MM, and PA than controls (at least, *p* < 0.01) (Table 1 and Figure 3). PA < 4° was in only 2 controls (2.67%) while was in 23 ACKD patients (30.66%) (*p* < 0.001) (data not shown). The PA was correlated positively with BCM% (ACKD patients: *r* = 0.54; controls: *r* = 0.84; both, *p* < 0.001), ICW% (ACKD patients: *r* = 0.93; controls: *r* = 0.76; both, *p* < 0.001), MM% (ACKD patients: *r* = 0.65; *p* < 0.001; controls: *r* = 0.35; *p* = 0.004) and s-CRP (ACKD patients: *r* = 0.31; *p =* 0.005; controls: *r* = 0.11; *p* = 0.36) but negatively with exchangeable Na/K (ACKD patients: *r* = −0.21; controls: *r* = −0.59; both, *p* < 0.001), ECW% (ACKD patients: *r* = −0.92; controls: *r* = −0.76; both, *p* < 0.001) and ECM (ACKD patients: *r* = −0.47; *p* < 0.001; controls: *r* = −0.38; *p* = 0.002).

Table 2 summarizes the clinical and biochemical parameters of 150 participants in the study. Patients with ACKD showed high concentrations of total protein, s-albumin, and s-CRP (at least, *p* < 0.05). Total cholesterol, lymphocyte count, hemoglobin, and PNI not differed in a significant manner between ACKD patients and controls. S-albumin was directly correlated with PNI (ACKD patients: *r* = 0.67; controls: *r* = 0.75; both, *p* < 0.001), and inversely with s-CRP (ACKD patients: *r* = −0.29 *p* < 0.001; controls: *r* = −0.70; *p* < 0.009). The median of PNI was in ACKD patients of 48.37 vs. 49.35 points in controls (*p* = 0.828). Only 2 ACKD patients and 2 controls (2.67%) had a PNI < 39 points. Significant differences were found only with the cut-off point of the PNI (<39 and ≥39 points) and s-CRP in ACKD patients (PNI < 39 points: 4.25 ± 5.16 and PNI ≥ 39 points: 0.95 ± 1.05) and controls (PNI < 39 points: 3.95 ± 3.46 and PNI ≥ 39 points: 0.29 ± 0.55) (both, *p* < 0.001) (data not shown).

### 3.2. Univariate Conditional Regression Analyses

Univariate conditional regression analyses showed that BIA-derived measurements, as well as hydration indicators (TBW, ECW, ICW), body composition measures (BCM, ECM, FM, FFM, MM,) and laboratory data (total protein, phosphorus, and s-CRP) were individually and independently associated between patients with ACKD and controls (dependent variable) (Table 3).

### 3.3. Multivariate Conditional Regression Analysis

The multivariate regression analysis demonstrated that certain nutritional and body composition measures such as TBW% (OR: 1.186), ECM (OR: 1.346), MM% (OR: 0.847), PA (0.058) such as well-recognized predictor biomarkers as s-albumin (OR: 0.475) and s-CRP (OR: 2.050) were significantly associated in cases and control after controlling for potential confounders (all at least, *p* < 0.05) (Table 4).

## 4. Discussion

The study results suggest that a miscellaneous combination of nutritional indicators, body composition measurements, and inflammatory markers were significant risk predictors in patients with ACKD compared with age- and sex-matched controls. In this study, older age, DM, overweight and abdominal obesity were classic CV risk factors in a Mediterranean cohort population. Among the universally recognized and accepted causes in the Western world, hypertension and DM are the main traditional risk factors involved in CVD [43,44], being the main cause leading to CKD in this cohort DM, is much more frequent in conditions of overweight or obesity such as those found in this study.

BMI is one of the most widely used and simpler measures of body adiposity. Overweight and obesity accounted for 76.0% of controls and 60.0% of ACKD patients, it is noteworthy that BMI in the univariate analysis had a significant protective effect (Table 3). In fact, these results exceed the combined prevalence of overweight and obesity in the Spanish population, estimated at 61.40% of Spaniard adults [45]. However, paradoxical benefits of being overweight/mildly obese termed “reverse epidemiology” had a U-shaped association with survival in ACKD patients and older adults [8,10]. A meta-analysis [9] concluded that overweight and obesity (BMI: 25–35 kg/m^2^) were associated with a lower risk of death in stages 3–5 CKD. In contrast, the usefulness of BMI as a single measure of body composition in CKD patients is limited, and hydration status and other measures of body composition must be taken into account. Conversely, the usefulness of BMI as a single measure of body composition in CKD patients is limited and should be considered in conjunction with hydration status and other measures of body composition. Overweight, obesity, and increased central fat distribution have been associated with microalbuminuria, reduced eGFR [46], and CVD [11].

Waist circumference (WC) and conicity index are clinically suggested to assess abdominal obesity and as a predictor of mortality in non-CKD subjects and CKD patients [21]. Additionally, in the general population, WC is strongly and independently associated with multiple traditional risk factors for CVD, including DM, hypertension, and dyslipidemia [47]. A WC ≥ 88 cm in females and ≥102 cm in males are proposed as health risk indicators concerning BMI [33]. In the current study, a significantly larger WC was found in matched females compared with males. In fact, in females, it was observed that among the 32 ACKD females (43.24%) and their 29 matched controls (*n* = 29; 39.1%), both had a WC above the threshold values, suggesting a high CV risk associated with central abdominal fat (*p* = 0.46). Likewise, in males, higher abdominal WC was non-significantly found in controls (*n* = 18; 23.7%) compared to ACKD patients (*n* = 13; 17.10%; *p* = 0.750). Similarly, the conicity index as a surrogate measure of CV risk and death was also found to increase in females and males in age-sex matched groups in a non-significant manner (*p* = 0.13). A study conducted on 1740 patients with stage 3 CKD [11] examined the ability of BMI and some anthropometric indicators of abdominal adiposity (WC, waist-to-height ratio, waist-to-hip ratio, and Conicity index). Central abdominal fat measured by any of the above indicators was more significant as a risk factor for CKD progression and as a predictor of CV risk than BMI, especially in older patients, where BMI tends to decrease with age [11].

The conicity index appears to be a good indicator of central fat distribution, both in patients with ACKD and in controls, as it identifies changes in body composition and thereby also allows comparisons to be made between individuals with different BW and height measurements [11]. However, in this cohort of ACKD patients, no significant association was detected between WC and conicity index in the univariate analysis among cases and controls, in agreement with the results published in a previous study [48]. Thus, findings from this study showed that both ACKD patients and controls had mean BMI values consistent with overweight in agreement with other studies [6,7,8,20] and high increased central abdominal fat, especially in females as a CV risk factor.

Overhydration, uremia-induced alterations in body composition, and inflammation are significant predictors of morbidity, poor prognosis, and mortality in patients with ACKD [7,49,50]. In this study, body composition measurements showed significantly that TBW, ECW, and ICW in ACKD patients differed from matched controls (Figure 2). Even with high mean s-albumin levels, patients with ACKD tended to be overhydrated or have fluid shifts between the ICW and ECW compartments, as well as higher mean exchangeable Na/K and ECM values than their control counterparts (Table 2). Furthermore, in ACKD patients, significantly lower BCM% and MM% were found, while a higher mean value of FFM% measures was also noted (Figure 3). These findings can be partially explained in terms of overhydration, as the decrease in BCM is the result of reduced ICW and/or muscle mass, whereas the increase in ECM is associated with an increased ECW balance. In addition, the univariate conditional regression analysis of this study showed that the above body composition measures (TBW, ECW, ECM, FFM) were significantly associated with nutritional risk factors in both cases and controls. Conversely, as expected, BCM% ICW%, FM% MM%, and PA were confirmed as significant protective factors in the univariate analysis. A subsequent multivariate conditional regression model further demonstrated that TBW was significantly associated as a risk factor between ACKD patients and controls. These results are aligned with previous studies [6,7,8] in which advanced age, overhydration secondary to impaired renal function, nutritional disorders, and/or uraemic disturbances in CKD stages 4–5 were also identified as causal factors for the above-mentioned conditions, similar to the current study.

Most notably, PA has been recognized as a reliable prognostic indicator of cellular integrity in elders, CKD patients, and other underlying diseases [17,49,51]. Lower mean PA values were seen in ACKD patients compared to matched controls. It has previously been reported that a lower PA cut-off point < 4° is an independent predictor of worse clinical outcomes and a predictor of long-term mortality in CKD and dialysis patients [17,51,52]. In the current study, a cut-off point was ≥4° in 69.3% of ACKD patients, being positively correlated with MM% and ICW% but negatively with ECM and s-CRP. These findings are consistent with the results discussed above, where PA can be used to detect any changes in nutritional status related to shifts in body composition due to muscle loss or fluid overload, often secondary to the effect of inflammation. In fact, the results obtained from the univariate and multivariate analysis showed that both PA and MM% were significantly and positively associated with better nutritional status among cases and controls (Table 4). Further studies in non-dialysis CKD patients are needed to define the PA cut-off point in order to diagnose nutritional disorders, adverse outcomes, and mortality at an earlier stage.

In CKD, specific markers such as serum concentrations of creatinine, uric acid, phosphorus, and potassium tend to be higher as kidney function declines, as observed in this study compared to controls. Higher mean serum uric acid levels, which were identified in previous studies as an independent CV risk marker in individuals with [53] and without CKD [54] were also significantly increased in the ACKD group, showing no association in the univariate analysis [11]. Interestingly, mean values for total protein and s-albumin levels were both significant and noticeably higher in patients with ACKD, whereas the concentration of s-CRP, a biomarker of inflammation, suggested signs of low-grade inflammation in patients with ACKD compared to matched controls.

S-albumin is one of the most studied markers related to nutritional status and mortality predictor in CKD patients [55,56]. In this study, s-albumin levels ≥ 4 g/dL were in 72.0% of ACKD patients with a mean s-CRP value of 0.802 ± 0.884 mg/dL. The elevated mean albumin concentration described in this study is similar to previous studies in patients with ACKD [6,8,20,28,50] but differs from other studies [7,38,49]. Findings from this study may be partly explained in this Mediterranean cohort. ACKD patients were medical and nutritionally followed up every 3 months or more frequently than usual care in a multidisciplinary ACKD unit. Nutritional recommendations based on protein intake were set at 0.6–0.8 g protein/kg/day and monitoring of sodium, potassium, and phosphorous food intakes was periodically supported by nutritional counseling in the framework of a Mediterranean diet pattern. Furthermore, inflammation is one of the major conditions contributing to nutritional disorders and is inversely related to s-albumin concentration [57]. Likewise, it is also important to note that s-albumin concentration is influenced not only by inflammation but also by other factors such as fluid overload. However, the above data suggest that chronic low-grade inflammation does not significantly interfere with s-albumin levels, as reported both in previous CKD research [16,42,43,44] and in this case-control study. In particular, s-albumin and s-CRP concentrations, as well as TBW, and ECM, were significantly associated in univariate and multivariate conditional regression analyses among cases and controls. An increase in albumin of 1 g/dL reduces the probability of nutritional risk by 52.5%, whereas s-CRP increases the probability of nutritional risk more than 2 times in this cohort. Data results confirm the inverse relationship between s-albumin and s-CRP in this cohort by multivariate conditional analysis.

The PNI has emerged as an independent predictor risk factor in elderly people with certain disease states [22,23,24,25,26,27,28,38,39,40,41,42]. Mean PNI values not differed in ACKD patients and controls, partly due to high mean s-albumin concentration and low grade of chronic inflammation found in this cohort. Thus, since s-albumin is involved in PNI, it was excluded from the conditional multivariate regression analysis in the study. Studies on PNI in older CKD are scarce. A retrospective study of 359 patients over 80 years of age with stage 3–4 CKD [28] showed that PNI was significantly associated with mortality and could potentially be useful in monitoring the nutritional status of elderly patients with CKD. The results of the study showed that a low PNI (<39 points) was associated with the onset of renal replacement therapy and an increased mortality rate [28]. By contrast, in the present study, the median PNI was ≥45 points (no nutritional risk) in both cases and controls. Collectively, these results demonstrate that this elderly Mediterranean cohort of ACKD patients had an adequate nutritional status when compared age-sex matched with their controls. Further studies are hence warranted to assess the diagnostic ability of PNI in elderly people with ACKD.

Some limitations and strengths should be taken into account in this study. Firstly, this is a single-center study conducted in an ACKD unit with small Mediterranean sample size. Diabetes mellitus was the main cause of CKD in cases while a lower proportion was found in controls. To date, there are a limited number of studies on elderly non-dialysis ACKD patients. However, to the best of our knowledge, this is the first case-control study in older adults with stages 4–5 ACKD, in which nutritional indicators, body composition measurements, and inflammation profile were jointly compared with age- and sex-matched controls. Nonetheless, the results obtained do not infer causality and may be biased by other biomarkers not analyzed in the present study. Secondly, BMI is a global indicator of adiposity that does not distinguish within adult age groups, body compartments (e.g., fat mass and muscle mass), or central fat distribution. In particular, in older adults and elderly patients with ACKD, it should be noted that BMI between 25–35 kg/m^2^ has been shown to reduce CV mortality in some meta-analyses [9,10]. Additionally, WC and conicity index were used in the present study as recommended measures of central abdominal fat in CKD [21]. Thirdly, BIA is one of the simplest methods used in clinical practice, although only one measure was evaluated. As further measures, this study used standardized Mediterranean anthropomorphic data and BIA-derived measurements together with nutritional-inflammatory markers such as s-albumin, and s-CRP as recognized risk indicators for nutritional disorders in ACKD. Fourth, dietary intake was not analyzed in the study, though patients with ACKD received regular dietary advice in the framework of the nutritional follow-up of the ACKD unit. In addition, pharmacological treatment was not recorded, although some drugs that could interfere with nutritional and inflammatory status (e.g., ONS, corticosteroids), were considered as an exclusion criterion for this study. Fourth, the PNI was used as the nutritional risk score (<39 points in this study), but several cut-off points have been proposed in different studies [27,39,42]. Lastly, the results from this study are not generalizable to early stages CKD or dialysis population.

## 5. Conclusions

In conclusion, this study highlights that the conjoint use of nutritional and inflammatory markers and BIA-derived measures appear as significant prognostic entities in patients with stage 4-5 older ACKD compared to matched controls. Overweight was the distinctive phenotype in both older adults and patients with ACKD, along with increased central abdominal fat, especially in women as a CV risk factor. Nutritional factors such as TBW, ECM, and s-CRP increased the likelihood of nutritional risk while independent predictors such as MM, PA, and s-albumin were shown to diminish the risk of nutritional disorders in ACKD. The results from the present study demonstrate the importance of regular nutritional care and monitoring as a preventive and/or therapeutic measure before starting dialysis. Further longitudinal studies in ACKD stages 4–5 are needed to assess the prognostic role of nutritional factors on both kidney disease progression and survival outcomes.

## Figures and Tables

**Table 1:** Demographic, clinical, and body composition parameters of the 150 participants in the study.

Variable	Total (*n* = 150)	ACKD (*n* = 75)	Controls (*n* = 75)	*p*-Value
Male, n (%)	76 (50.66)	38 (25.33)	38 (25.33)	
Age (yrs)	80.07 ± 6.62	79.72 ± 6.69	80.43 ± 6.57	0.450
DM n; (%)	22 (14.66)	15 (20.00)	7 (9.33)	<0.001
BW (%)	69.46 ± 13.04	69.23 ± 12.71	69.68 ± 13.43	0.830
SBW (%)	111.38 ± 21.51	110.05 ± 18.95	112.71 ± 23.84	0.560
BMI (kg/m^2^)	27.66 ± 5.61	26.58 ± 4.64	28.73 ± 6.28	0.018
WC (cm)	99.52 ± 12.52	97.83 ± 12.41	101.26 ± 12.48	0.095
Conicity index	1.38 ± 0.11	1.37 ± 0.11	1.40 ± 0.12	0.130
TSF (%)	138.68 ± 82.15	131.55 ± 71.77	145.81 ± 91.29	0.290
MAMC (%)	147.97 ± 29.88	148.95 ± 28.65	146.99 ± 31.23	0.159
TBW (L)	36.25 ± 6.43	37.11 ± 5.90	35.37 ± 6.87	0.100
ECW (L)	17.84 ± 4.01	20.11 ± 3.68	15.54 ± 2.86	<0.001
ICW (L)	17.74 ± 3.82	17.12 ± 3.75	19.22 ± 3.63	0.010
Exchangeable Na/K	1.00 ± 0.25	1.10 ± 0.29	0.90 ± 0.17	<0.001
ECM (kg)	1.12 ± 0.50	1.43 ± 0.46	0.80 ± 0.29	<0.001
BCM (kg)	22.06 ± 5.56	19.49 ± 4.36	24.63 ± 5.46	<0.001
FM (kg)	24.87 ± 9.82	22.78 ± 8.35	27.01 ± 10.77	0.008
FFM (kg)	44.94 ± 7.53	46.02 ± 7.15	43.84 ± 7.78	0.078
MM (kg)	29.08 ± 8.83	25.69 ± 6.56	32.62 ± 9.53	<0.001
PA (°)	5.26 ± 1.00	4.51 ± 0.86	6.09 ± 0.97	<0.001

*p*-Values are based on Chi-square or Student’s *t*-tests. BCM, body cell mass; BMI, body mass index; DM, diabetes mellitus; ECM, extracellular mass; ECW, extracellular water; FFM, fat-free mass; FM, fat mass; ICW, intracellular water; MAMC, mid-arm muscle circumference; MM, muscle mass; PA, phase angle; SBW, standard body weight; TSF, triceps skinfold thickness; WC, waist circumference.

**Table 2:** Clinical and biochemical parameters characteristics of 150 participants in the study.

Variable	Total (*n* = 150)	ACKD (*n* = 75)	Controls (*n* = 75)	*p*-Value
*e-GFR (mL/min/1.73 m^2^)	48.23 ± 35.07	14.44 ± 7.11	92.03 ± 10.60	<0.001
s-Creatinine (mg/dL)	2.38 ± 1.73	3.82 ± 1.33	0.95 ± 0.28	<0.001
Uric acid (mg/dL)	6.15 ± 1.97	7.40 ± 1.85	4.91 ± 1.14	<0.001
s-Calcium (mg/dL)	9.25 ± 1.39	9.51 ± 0.81	8.11 ± 2.50	<0.001
s-Phosphorous (mg/dL)	4.40 ± 0.67	4.69 ± 0.77	4.12 ± 0.39	<0.001
s-Potassium (mEq/L)	4.75 ± 0.57	4.85 ± 0.63	4.64 ± 0.48	0.054
s-Cholesterol (mg/dL)	170.03 ± 34.21	174.03 ± 29.70	166.03 ± 37.97	0.153
Total proteins (mg/dL)	6.72 ± 0.67	7.02 ± 0.58	6.40 ± 0.61	<0.001
s-Albumin (mg/dL)	4.04 ± 0.28	4.09 ± 0.27	3.98 ± 0.28	0.030
s-CRP (mg/dL)	0.71 ± 1.15	1.04 ± 1.31	0.38 ± 0.84	<0.001
Hemoglobin (g/dL)	12.50 ± 1.22	12.43 ± 1.10	12.58 ± 1.33	0.466
Lymphocytes count (×10^3^/mm^3^)	1823.72 ± 633.07	1730.14 ± 702.54	1916.05 ± 545.23	0.073
PNI (points)	49.35 ± 4.4 mediana	49.27± 4.68	49.42 ± 4.13	0.828

*p*-Values are based on Chi-square or Student’s *t*-tests. *e-GFR, estimated glomerular filtrate rate was measured by 2021 CKD-EPI Creatinine equation [29]. PNI, prognosis nutritional index; s-CRP, serum C-reactive protein.

**Table 3:** Univariate conditional regression analysis in cases and controls.

Variable	OR	St Error	CI95%	*p*-Value
BMI (kg/m^2^)	0.933	0.028	0.285 to 0.088	0.027
Conicity index	0.100	0.153	0.005 to 2.010	0.133
BCM (%)	0.737	0.057	0.632 to 0.863	<0.001
ECM (kg)	1.392	0.109	1.193 to 1.625	<0.001
TBW (%)	1.078	0.030	1.020 to 1.138	<0.001
ECW(%)	1.437	0.123	1.213 to 1.701	<0.001
ICW (%)	0.686	0.062	0.573 to 0.822	<0.001
FM (%)	0.941	0.021	0.904 to 0.984	0.008
FFM (%)	1.077	0.025	1.029 to 1.127	<0.001
MM (%)	0.912	0.024	0.866 to 0.961	0.001
PA (°)	0.036	0.037	0.004 to 0.278	<0.001
Total proteins (g/dL)	7.311	3.456	2.894 to 18.467	<0.001
Phosphorous (mg/dL)	5.338	2.098	2.470 to 11.536	<0.001
s-Albumin (g/dL)	0.289	0.411	0.072 to 1.149	0.072
s-CRP (mg/dL)	2.167	0.556	1.310 to 3.584	0.003
Lymphocytes count (×10^3^/mm^3^)	0.999	0.0002	0.998 to 1.000	0.077

*p*-Values are based on univariate conditional regression analysis using cases and controls as a *dummy* variable. CI95%, confidence interval 95%; OR, odds ratio; St error, standard error. BCM, body cell mass; BMI, body mass index; ECM, extracellular mass; ECW, extracellular water; FFM, fat-free mass; FM, fat mass; ICW, intracellular water; MM, muscle mass; PA, phase angle; s-CRP, serum C-reactive protein; TBW, total body water.

**Table 4:** Multivariate conditional regression analysis in cases and controls.

Variable	OR	St Error	CI95%	*p*-Value
Total body water (%)	1.186	0.061	1.076 to 1.314	0.001
Extracellular mass (kg)	1.346	0.106	1.153 to 1.572	<0.001
Muscle mass (%)	0.847	0.037	0.776 to 0.922	<0.001
Phase angle (°)	0.058	0.059	0.008 to 0.4201	0.005
s-Albumin (g/dL)	0.475	0.142	0.263 to 0.856	0.013
s-CRP (mg/dL)	2.050	0.577	1.180 to 3.561	0.011

*p*-Values are based on multivariate conditional regression analysis using cases and controls as a *dummy* variable. CI95%, confidence interval 95%: OR, odds ratio; St error, standard error. s-CRP, serum C-reactive protein.

## Data Availability

Not applicable.
